# PMA synergistically enhances apicularen A-induced cytotoxicity by disrupting microtubule networks in HeLa cells

**DOI:** 10.1186/1471-2407-14-36

**Published:** 2014-01-22

**Authors:** Kang-Sik Seo, Jong-Seok Kim, Ji-Hoon Park, Kyoung-Sub Song, Eun-Jin Yun, Jong-Il Park, Gi Ryang Kweon, Wan-Hee Yoon, Kyu Lim, Byung-Doo Hwang

**Affiliations:** 1 Department of Biochemistry, College of Medicine, Chungnam National University, Daejeon, Korea; 2 Cancer Research Institute, Chungnam National University, Daejeon, Korea; 3 Infection Signaling Network Research Center, Chungnam National University, Daejeon, Korea

**Keywords:** PMA, Apicularen A, PKCα, Cell death, Microtubule disruption

## Abstract

**Background:**

Combination therapy is key to improving cancer treatment efficacy. Phorbol 12-myristate 13-acetate (PMA), a well-known PKC activator, increases the cytotoxicity of several anticancer drugs. Apicularen A induces cytotoxicity in tumor cells through disrupting microtubule networks by tubulin down-regulation. In this study, we examined whether PMA increases apicularen A-induced cytotoxicity in HeLa cells.

**Methods:**

Cell viability was examined by thiazolyl blue tetrazolium (MTT) assays. To investigate apoptotic potential of apicularen A, DNA fragmentation assays were performed followed by extracting genomic DNA, and caspase-3 activity assays were performed by fluorescence assays using fluorogenic substrate. The cell cycle distribution induced by combination with PMA and apicularen A was examined by flow cytometry after staining with propidium iodide (PI). The expression levels of target proteins were measured by Western blotting analysis using specific antibodies, and α-tubulin mRNA levels were assessed by reverse transcription polymerase chain reaction (RT-PCR). To examine the effect of combination of PMA and apicularen A on the microtubule architecture, α-tubulin protein and nuclei were visualized by immunofluorescence staining using an anti-α-tubulin antibody and PI, respectively.

**Results:**

We found that apicularen A induced caspase-dependent apoptosis in HeLa cells. PMA synergistically increased cytotoxicity and apoptotic sub-G_1_ population induced by apicularen A. These effects were completely blocked by the PKC inhibitors Ro31-8220 and Go6983, while caspase inhibition by Z-VAD-fmk did not prevent cytotoxicity. RNA interference using siRNA against PKCα, but not PKCβ and PKCγ, inhibited cytotoxicity induced by combination PMA and apicularen A. PMA increased the apicularen A-induced disruption of microtubule networks by further decreasing α- and β-tubulin protein levels in a PKC-dependent manner.

**Conclusions:**

These results suggest that the synergy between PMA and apicularen A is involved by PKCα activation and microtubule disruption, and that may inform the development of novel approaches to treat cancer.

## Background

Apicularen A is a potent cytotoxic macrolide isolated from the myxobacterial genus *Chondromyces*[1] that induces apoptosis in several cancer cell lines such as the murine RAW 264.7 leukemia macrophage line and the human HL-60 promyelocytic leukemia cell line [2-4]. In addition, apicularen A induces apoptotic cell death in human HM7 colon cancer cells by up-regulating Fas ligand and disrupting microtubule architecture [5].

Protein kinase C (PKC) is a serine/threonine protein kinase family; PKC family members are classified into three major groups based on their activation pathways [6]: classical PKC isotypes (α, βI, βII and γ) are activated by diacylglycerol (DAG) and calcium, novel PKC isotypes (δ, ϵ, η and θ) are activated by DAG, and atypical PKC isotypes (ζ and ι/λ) are not regulated by DAG or calcium [7]. PKCs are associated with the regulation of cellular processes such as cell proliferation, differentiation and cell death [7]; however, the role of each PKC isotype in cellular processes, especially cell death, is controversial. For example, PKCδ and PKCθ are involved in apoptotic cell death through caspase-3-mediated proteolytic activation [8,9], while PKCϵ and PKCζ are involved in cell survival [10,11]. In addition, PKC activators such as phorbol 12-myristate 13-acetate (PMA) or bryostatin 1 can increase or decrease anticancer drug activity depending on the drugs and cell lines tested [12-16]. Thus, the functional significance of PKCs in cell death mechanisms remains elusive.

Microtubules are an important cytoskeletal component formed by the polymerization of α- and β-tubulin heterodimers [17]; they regulate several cellular processes including the maintenance of cell shape, motility, transport, organelle distribution and chromosome segregation during mitosis [18]. Since cancer cells proliferate more rapidly than normal cells, microtubules are considered a suitable therapeutic target and several anticancer drugs inhibit their function [19]. For example, *Vinca* alkaloids inhibit tumor cell proliferation by inducing the depolymerizaiton of microtubules [20], and taxanes induce apoptosis by promoting microtubule assembly [21]. Apicularen A disrupts microtubule networks by inhibiting tubulin synthesis [5]. Efforts to develop more effective cancer therapy combinations with microtubule-interfering agents are underway. The finding that PMA increases the antitumor activity of paclitaxel, a chemotherapeutic agent that inhibits tubulin polymerization, *in vitro* and in a xenograft model of prostate cancer [22] prompted us to test whether PMA increases apicularen A-induced cell death. The results of the present study demonstrate that PMA-mediated PKCα activation strongly increases apicularen A-induced apoptotic cell death and disruption of microtubule networks in HeLa cells.

## Methods

### Cell culture

Human HeLa cervical cancer cells (ATCC, Rockville, MD) were cultured in Dulbecco’s modified Eagle’s medium supplemented with 10% fetal bovine serum and antibiotics. Cells were maintained at 37°C, 5% CO_2_ and 95% air.

### Antibodies and chemicals

Apicularen A was provided by Dr. Ahn (Division of Ocean Science, Korea Maritime University, Busan, Korea) and dissolved in dimethyl sulfoxide. Phorbol 12-myristate 13-acetate (PMA), thiazolyl blue tetrazolium bromide (MTT), anti-α-tubulin and anti-β-tubulin antibodies were purchased from Sigma (St Louis, MO, USA). Anti-PARP and anti-actin antibodies were purchased from Santa Cruz Biotechnology (Santa Cruz, CA, USA). Anti-caspase-3 antibody was purchased from R&D Systems (Wiesbaden, Germany). Z-VAD-fmk, Ro31-8220 and Go6983 were purchased from Calbiochem (San Diego, CA, USA). All other reagents were molecular biology grade.

### Cell viability assay

Cell viability was assessed by thiazolyl blue tetrazolium (MTT) assay. Exponentially growing cells were exposed to apicularen A in the presence or absence of PMA for 24 and 48 hours. MTT solution was added to each well (0.5 mg/ml) and incubated for 2 hours. Cell viability was assessed by measuring the absorbance at 570 nm in an ELISA plate reader.

### DNA fragmentation assay

The cells were lysed using buffer containing 300 mM Tris–HCl (pH 7.5), 100 mM NaCl, 10 mM EDTA, 200 mM sucrose and 0.5% SDS. Intracellular DNA was extracted with phenol/chloroform (1:1) and chloroform/isoamylalcohol (24:1). DNA was precipitated and digested in 10 mM Tris–HCl (pH 8.0), 1 mM EDTA and 40 μg/ml RNase A for 1 hour at 37°C. Then, DNA (10 μg) was resolved by electrophoresis in a 1.2% agarose gel supplemented with ethidium bromide (0.2 μg/ml), and DNA fragmentation was examined by ultraviolet transillumination.

### Caspase-3 activity assay

Cell extracts were prepared by suspending 2 × 10^6^ HeLa cells in 100 μL TTE buffer [10 mM Tris–HCl (pH 8.0), 0.5% Triton X-100, 10 mM EDTA] on ice for 30 min, and then centrifuging at 15,000 × *g* for 10 minutes at 4°C. Lysates (30 μg total protein in 10 μl) were mixed with 90 μl assay buffer [20 mM HEPES (pH 7.5), 10% glycerol, 2 mM DTT] containing 40 μM Ac-DEVD-AFC. Caspase-3 activity was measured at 37°C using a spectrofluorometric plate reader (Perkin-Elmer LS-50B., Foster City, CA, USA) in kinetic mode using excitation and emission wavelengths of 400 nm and 505 nm.

### Western blotting analysis

HeLa cells were lysed in buffer containing 50 mM Tris–HCl (pH 7.5), 150 mM NaCl, 1% nonidet P-40, 0.5% deoxycholate, 0.1% SDS and protease inhibitor cocktail (Roche Applied Science, Mannheim, Germany). Cell lysates were subjected to SDS-PAGE and transferred onto nitrocellulose (Pall Life Sciences, Port Washington, NY, USA) or PVDF membranes (Millipore, Woburn, MA, USA). The membranes were first probed with primary antibodies and then with HRP-conjugated secondary antibodies (Calbiochem), and the proteins were detected using the ECL system (Amersham Biosciences Corp., Piscataway, NJ, USA).

### Cell cycle analysis

HeLa cells exposed to apicularen A in the presence or absence of PMA were washed with phosphate buffered saline (PBS) and fixed in 70% ethanol at −20°C overnight. Before analysis, cells were centrifuged and incubated with propidium iodide (50 μg/ml) supplemented with RNase A (1 mg/ml) for 30 minutes at room temperature. The relative DNA content was measured by flow cytometry using a Becton-Dickinson FACSort and by manual gating using CellQuest software.

### Reverse transcription-polymerase chain reaction (RT-PCR)

The mRNA level of α-tubulin was measured by RT-PCR. Total RNA was isolated using the TRIzol® reagent (Invitrogen, Karlsruhe, Germany) according to the manufacturer’s instructions. Complementary DNA (cDNA) was synthesized using AMV reverse transcriptase at 42°C for 1 hour. The mixture was then boiled for 5 minutes to inactivate reverse transcriptase and quickly chilled on ice. The cDNAs were amplified by RT-PCR using the HiPi Plus PCR master mix (Elpis Biotech, Korea). PCR products were separated on 1.2% agarose gels with ethidium bromide (0.2 μg/mL), and amplification products were examined by ultraviolet transillumination.

### Immunofluorescence assay

HeLa cells were seeded onto glass coverslips and were exposed to apicularen A in the presence or absence of PMA. The cells were washed twice with PBS, permeabilized with 0.25% triton X-100 and 0.5% glutaraldehyde for 1 minute at room temperature, and then fixed with 1% glutaraldehyde for 10 minutes before overnight incubation with anti-α-tubulin antibody diluted 1:500. After washing three times in PBS containing 0.1% Tween-20 (PBS/T), cells were incubated for 1 hour with secondary antibody (Alexa Fluor 488 goat anti-mouse IgG diluted 1:400) in the dark. After washing five times, cells were stained with 20 μg/ml propidium iodide and 1 mg/ml RNase A for 20 minutes at room temperature. Microtubules and nuclei were observed using an FV-500 fluorescence microscope (Olympus, Dulles, VA, USA). The fluorescence intensity was quantified using image J software.

### Statistical analysis

Results are expressed as the means ± SE. Statistical significance was assessed using the Student’s *t*-test and analysis of variance (ANOVA). *P* < 0.05 was considered to be significant. The combined effect of PMA and apicularen A (combination index) was calculated using the formula %AB/%A × %B, where A and B are the effects of each individual agent and AB is the effect of the combination. When the ratio (combination index) is 1 the effect is considered additive; when the combination index is significantly greater than or less than 1, the effect is considered subadditive (negative synergism) or supraadditive (positive synergism), respectively [23]. Statistical significance value of the combination index was compared with the additive combination index of 1 by one-sided Student’s t-test.

## Results

### Apicularen A induces cytotoxicity in HeLa cells

**Figure 1:**
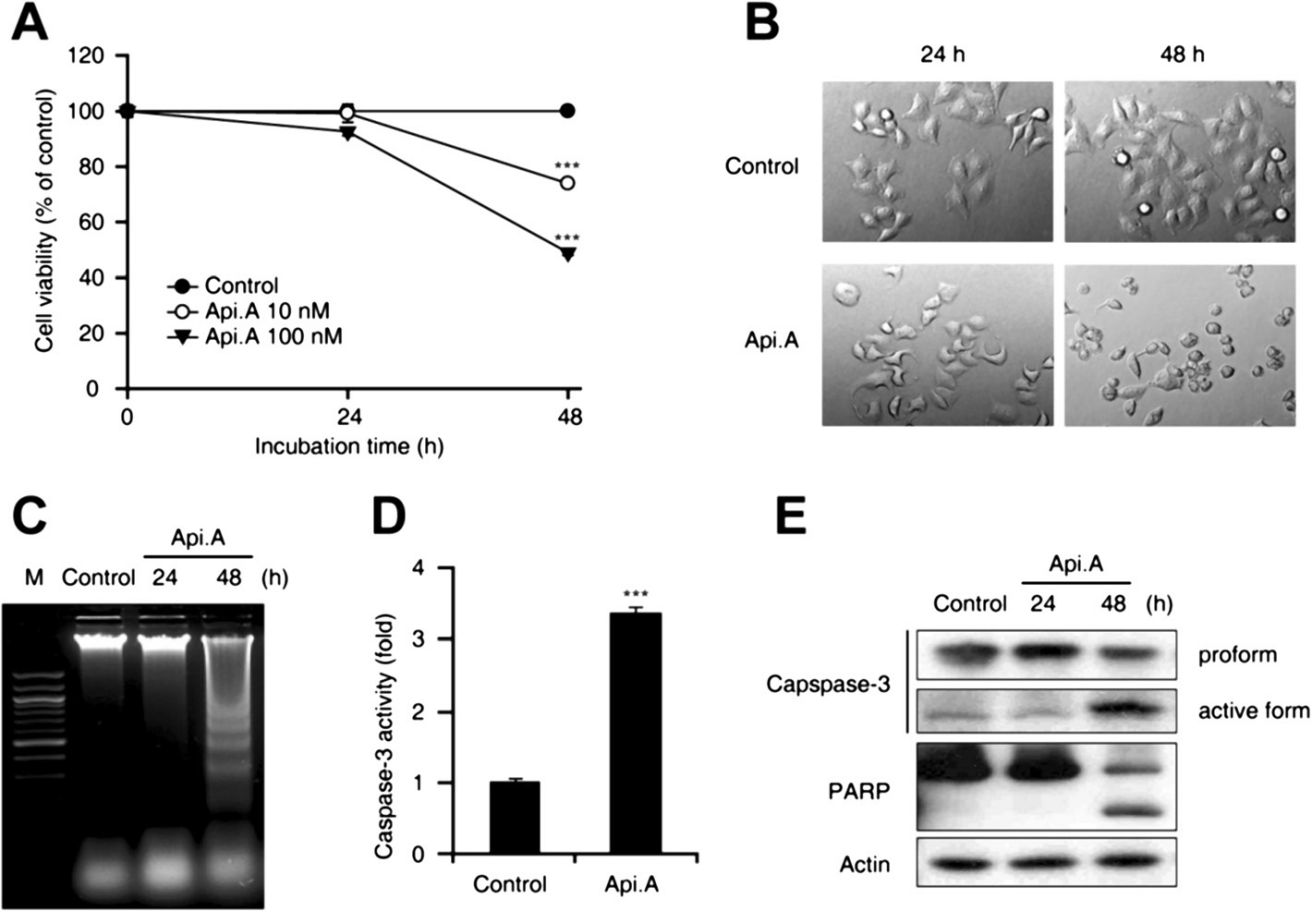
**Apicularen A induces apoptotic cell death in HeLa cells. A**. HeLa cells were exposed to 10 or 100 nM apicularen A. Cell viability was analyzed by MTT assay. **B**. HeLa cells were exposed to 100 nM apicularen A for 24 and 48 hours. Phase contrast images (X200) were acquired using an inverted microscope. **C**. Total genomic DNA of apicularen A-treated HeLa cells was extracted and analyzed by agarose gel electrophoresis. **D**. Caspase-3 activity of apicularen A-treated HeLa cells was assessed at 48 hours using the caspase-3 fluorogenic peptide substrate DEVD-AFC. **E**. HeLa cells were exposed to 100 nM apicularen A. Cell lysates were subjected to Western blotting with anti-caspase-3 and anti-PARP antibodies. All error bars indicate ± SEM. ***P < 0.001.

The effect of apicularen A on HeLa cell growth was performed. Apicularen A decreased cell viability in a concentration and time-dependent manner (Figure 1A). In addition, suspended HeLa cells exposed to apicularen A exhibited membrane blebbing, nuclear condensation and shrinkage of the cytoplasm (Figure 1B). To investigate whether these morphological changes were caused by apoptosis, genomic DNA was purified and analyzed for fragmentation. As shown in Figure 1C, apicularen A induced DNA fragmentation at 48 hours. Since caspase-3 plays a crucial role in apoptotic cell death by cleaving poly (ADP-ribose) polymerase (PARP) to suppress the DNA repair pathway [24], we tested the potential involvement of caspase-3 in apicularen A-induced cell death by measuring caspase-3 activity and the PARP cleavage. HeLa cells exposed to apicularen A exhibited a 3-fold increase in caspase-3 activity compared to control cells (Figure 1D). In addition, apicularen A increased the active form of caspase-3 and the cleaved form of PARP (Figure 1E). Taken together, these results indicate that apicularen A induces apoptotic cell death in HeLa cells.

### PMA increases apicularen A-induced cytotoxicity in HeLa cells

**Figure 2:**
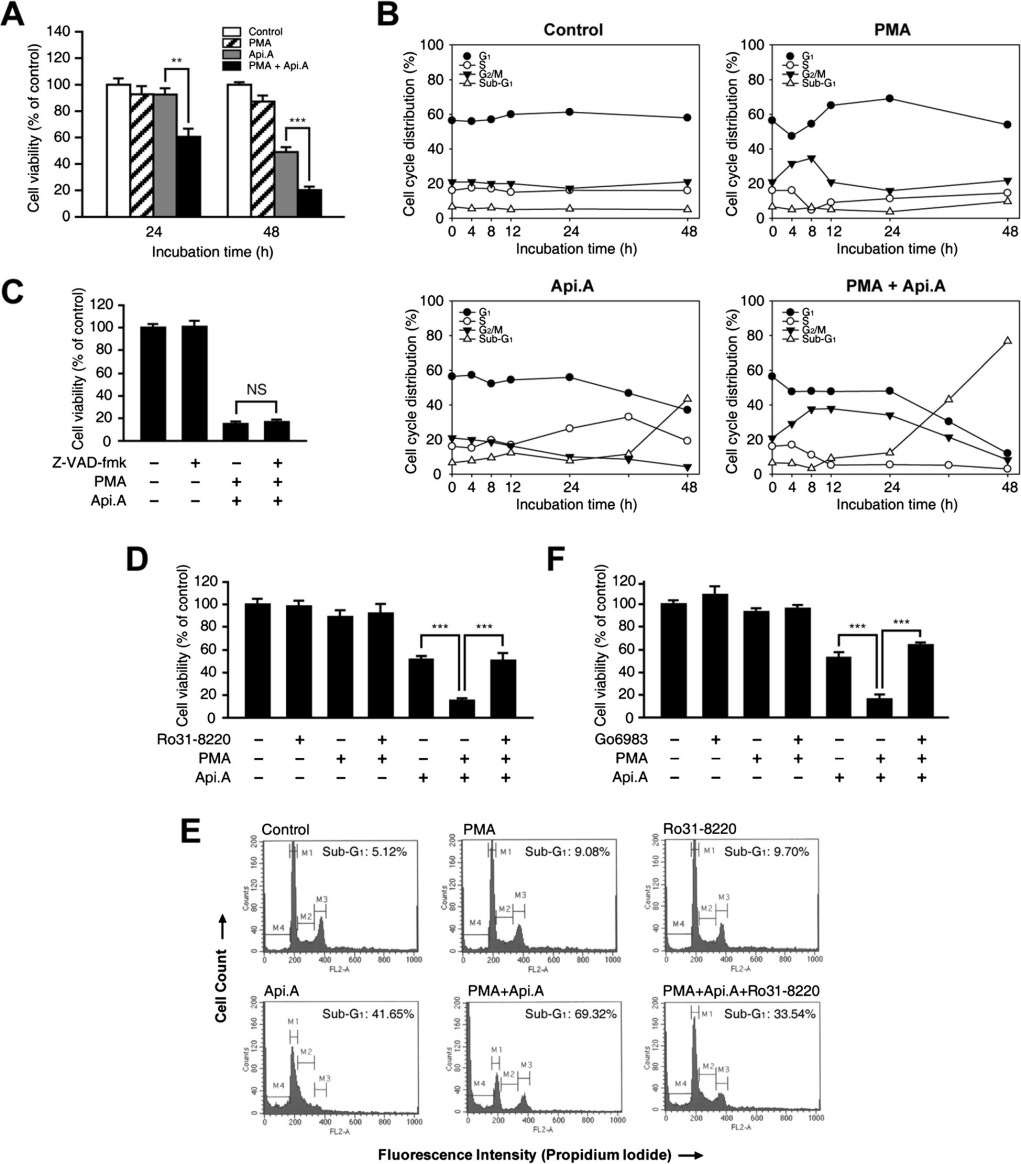
**PMA increases apicularen A-induced cytotoxicity in HeLa cells. A**. HeLa cells were exposed to 20 nM PMA and 100 nM apicularen A for 24 and 48 hours. Cell viability was analyzed by MTT assay. **B**. HeLa cells were exposed to 100 nM apicularen A in the presence or absence of 20 nM PMA. DNA content was analyzed by flow cytometry. **C**. HeLa cells were pretreated with 10 μM Z-VAD-fmk and then exposed to 100 nM apicularen A in the presence or absence of 20 nM PMA for 48 hours. Cell viability was analyzed by MTT assays. **D**. HeLa cells were pretreated with 10 μM Ro31-8220 and then treated with 100 nM apicularen A in the presence or absence of 20 nM PMA for 48 hours. MTT assays were performed to evaluate cell viability. **E**. DNA content described in **D** was measured by flow cytometry. **F**. HeLa cells were pretreated with 2 μM Go6983 and then exposed to 100 nM apicularen A in the presence or absence of 20 nM PMA for 48 hours. All error bars indicate ± SEM. **P < 0.01, ***P < 0.001.

The role of PKC in the mechanism of action of antitumor agents is controversial since PKC synergizes with some agents and antagonizes others [12,14,15,25,26]. PMA was used to assess the effect of PKC on apicularen A-induced cytotoxicity. PMA has a similar chemical structure to DAG and activates PKCs by interacting with the DAG binding site [27]. HeLa cells were exposed to 100 nM apicularen A and 20 nM PMA; as shown in Figure 2A and Table 1, PMA synergistically increased the cytotoxicity of apicularen A in a time-dependent manner. This finding was supported by time-lapse video microscopy, showing that combination of PMA and apicularen A strongly induced cell death to a greater extent than apicularen A alone (Additional file 1: Movie S1). Next, flow cytometry was used to assess the potential effect of PMA on the cell cycle. Forty percent of apicularen A-treated cells were apoptotic (sub-G_1_ peak) at 48 hours, while no apoptosis was detected in control or PMA-treated cells (Figure 2B). In addition, 80% of the cells exposed to the combination of apicularen A and PMA were apoptotic at 48 hours, indicating that PMA increased apicularen A-induced apoptotic cell death. Since apicularen A induced apoptotic cell death through caspase activation (Figure 1E), we investigated whether the cytotoxicity induced by the combination of PMA and apicularen A also depended on caspase activation. The pan-caspase inhibitor Z-VAD-fmk did not block the cytotoxicity induced by the drug combination (Figure 2C), indicating that the synergy is caspase-independent. Since PMA is involved in several PKC-independent cellular processes associated with cell proliferation and differentiation [28-30], the role of PKC activation on the synergy with apicularen A was tested by exposing cells to PKC inhibitor Ro31-8220 before adding PMA and apicularen A. Ro31-8220 showed no cytotoxicity in HeLa cells at 48 hours, and pretreatment with Ro31-8220 completely blocked the synergistic apoptotic activity of PMA and apicularen A (Figure 2D and 2E). The PKC inhibitor Go6983 also suppressed the effect of PMA on apicularen A-induced cytotoxicity (Figure 2F). These results suggest that PKC activation increases apicularen A-induced apoptotic cell death.

**Table 1:** Analysis of the interaction between the inhibitory effect of PMA in combination with apicularen A on the viability of HeLa cells

**Combination index**	**p value**	**Interaction**
0.113 ± 0.004	0.001	Synergy

The combination index was calculated using the formula %AB/%A × %B, as described under “Methods”. The value indicate ± SEM.

### PKCα mediates the effect of PMA on apicularen A-induced cytotoxicity

**Figure 3:**
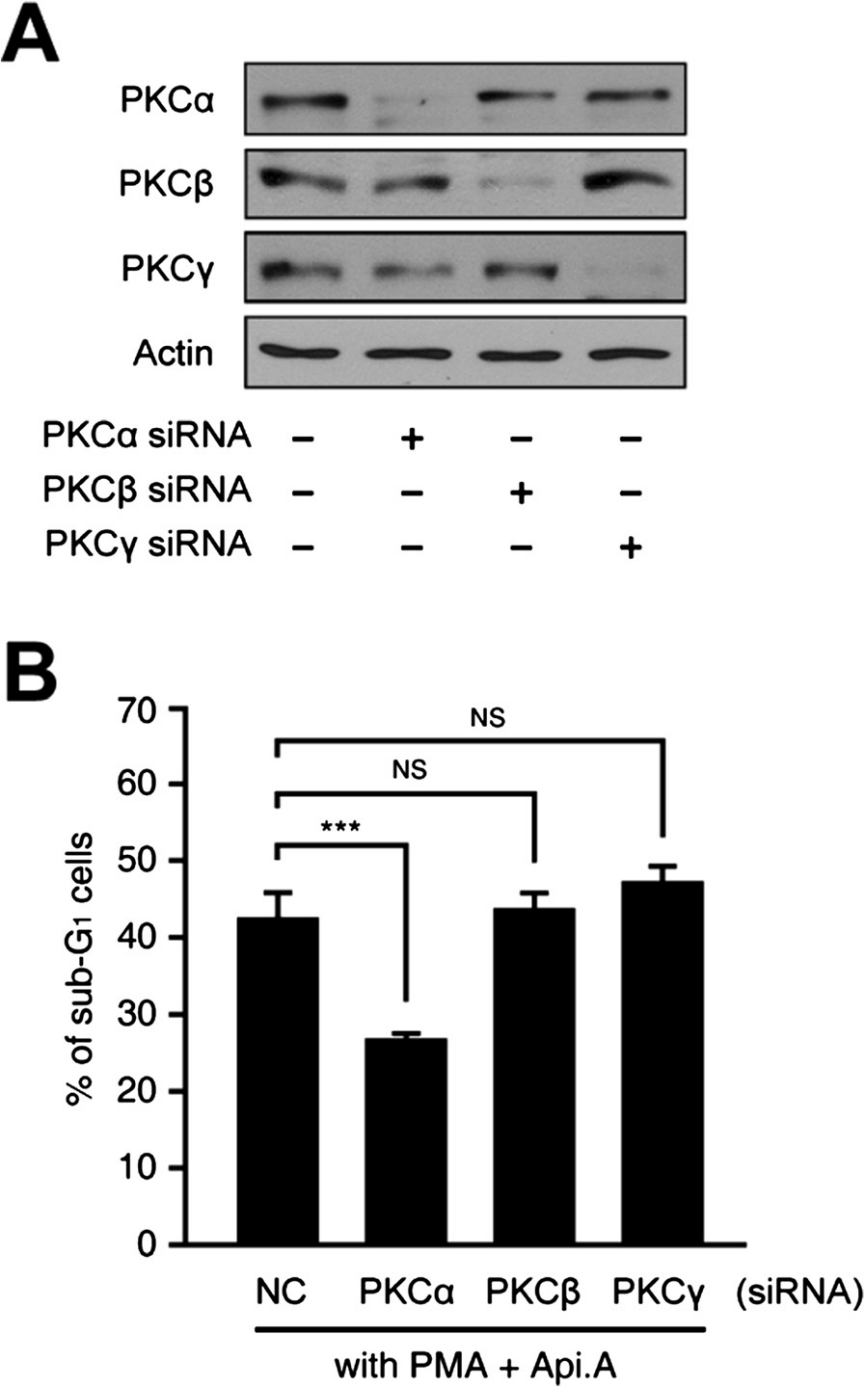
**PMA increases apicularen A-induced cell death in HeLa cells by activating PKCα. A**. HeLa cells were transfected with siRNAs against PKCα, PKCβ and PKCγ, and incubated for 48 hours. PKCα, PKCβ and PKCγ protein levels were assessed by Western blotting. **B**. HeLa cells expressing siRNAs against PKCα, PKCβ and PKCγ were exposed to 20 nM PMA and 100 nM apicularen A for 36 hours. DNA content was analyzed by flow cytometry. Error bars indicate ± SEM. ***P < 0.001.

To identify which PKC isotype is involved in the increase in apicularen A-induced cell death observed in the presence of PMA, cells were pretreated with siRNAs specific for individual PKCs and exposed to PMA and apicularen A. Since PMA activates the classical and novel PKC isotypes and Go6983 inhibits PKCα, β, δ, γ and ζ [27,31], specific siRNAs against PKCα, β and γ were needed; the isotype-specificity of the knockdown was confirmed by transient transfection and Western blotting in HeLa cells (Figure 3A). As shown in Figure 3B, knockdown of PKCα, but not that PKCβ or PKCγ, significantly decreased the apoptosis induced by the combination of PMA and apicularen A. These results demonstrate that PKCα mediates the synergistic effect of PMA on apicularen A-induced cell death in HeLa cells.

### PMA increases apicularen A-mediated tubulin down-regulation

**Figure 4:**
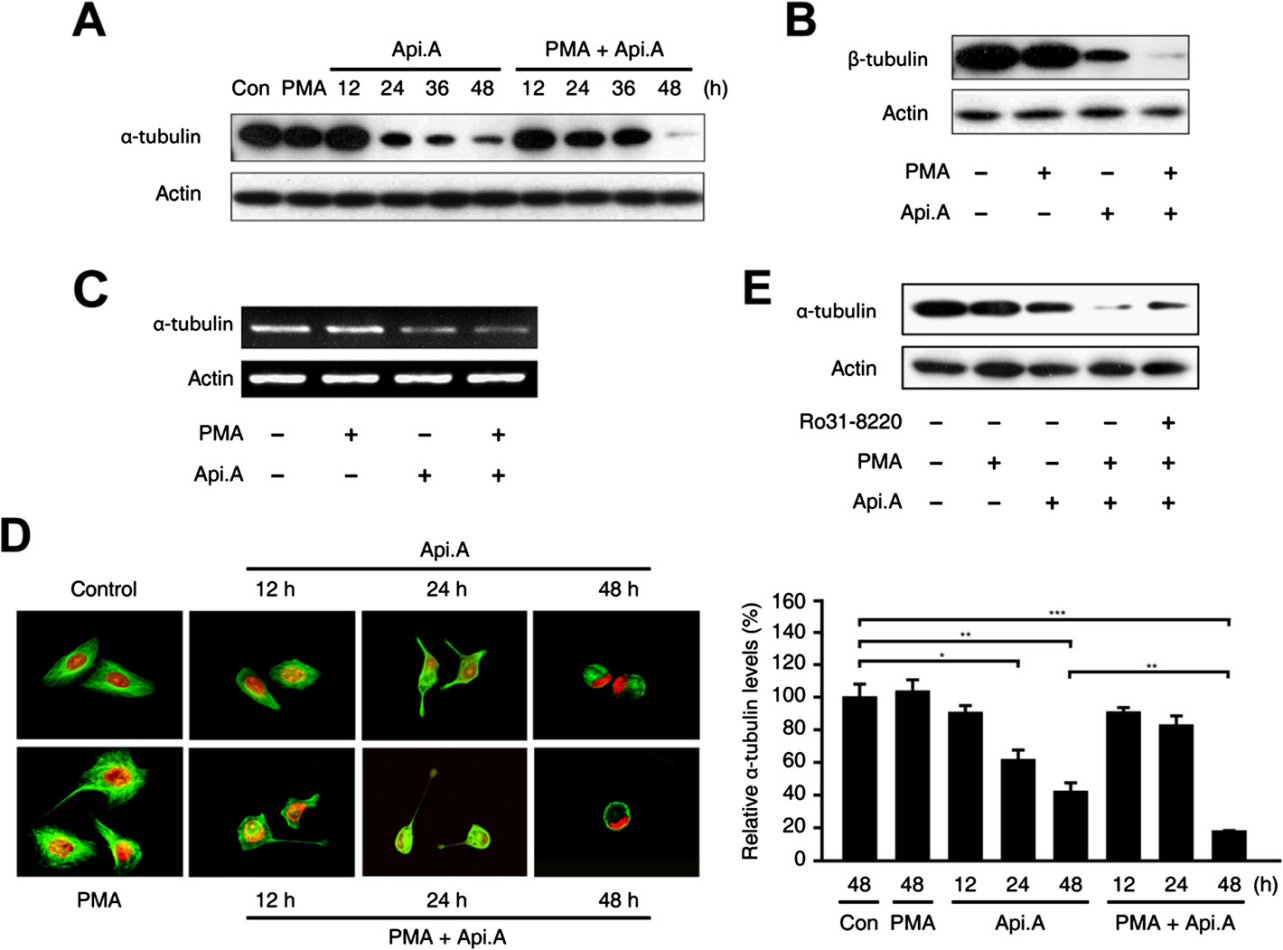
**PMA increases apicularen A-mediated tubulin down-regulation. A**. HeLa cells were exposed to 100 nM apicularen A in the presence or absence of 20 nM PMA. α-tubulin protein levels were analyzed by Western blotting. **B**. HeLa cells were exposed to 100 nM apicularen A in the presence or absence of 20 nM PMA for 48 hours. β-tubulin protein levels were analyzed by Western blotting. **C**. HeLa cells were exposed to 100 nM apicularen A in the presence or absence of 20 nM PMA for 48 hours. α-tubulin mRNA levels were analyzed by RT-PCR. **D**. HeLa cells were exposed to 100 nM apicularen A in the presence or absence of 20 nM PMA. Cellular microtubule networks and nuclei were observed using an Olympus FV-500 fluorescence microscope. The quantified levels of α-tubulin under each of the indicated conditions are shown in the histograms on the right. Error bars indicate ± SD. *P < 0.05; **P < 0.01; ***P < 0.001. **E**. HeLa cells were pretreated with 10 μM Ro31-8220 and then exposed to 100 nM apicularen A in the presence or absence of 20 nM PMA for 48 hours. α-tubulin protein levels were analyzed by Western blotting.

We previously reported that the mechanism of apicularen A-induced apoptotic cell death in human HM7 colon cancer cells partially involved a decrease in intracellular tubulin levels [5]. Thus, we investigated whether PMA increases apicularen A-induced apoptotic cell death by further down-regulating tubulin. Apicularen A decreased total α-tubulin protein levels in a time-dependent manner (Figure 4A). At 48 hours, the combination of PMA and apicularen A decreased α-tubulin protein levels to a greater extent than apicularen A alone, while PMA alone had no effect. Similar results were obtained for β-tubulin (Figure 4B). Since apicularen A down-regulates tubulin levels by decreasing tubulin mRNA levels in HM7 cells [5], tubulin mRNA levels were assessed by RT-PCR in cells exposed to PMA and apicularen A. PMA did not affect α-tubulin mRNA levels in apicularen A-treated cells (Figure 4C). Microtubule architecture was assessed by immunofluorescence using anti-α-tubulin antibody and propidium iodide (PI). PMA-treated cells exhibited similar microtubule architecture to control cells. By contrast, apicularen A induced irregular microtubule networks and nuclear localization, and reduced α-tubulin protein levels. In addition, PMA further increased the effect of apicularen A on the microtubule networks and on α-tubulin levels (Figure 4D). Given that PMA increases apicularen A-induced cell death by increasing PKC activity, the possibility that PKC activation might also be responsible for the effect of PMA and apicularen A on tubulin protein levels was considered. HeLa cells were pretreated with Ro31-8220 and then exposed to apicularen A in the presence or absence of PMA. As expected, inhibition of PKC activity by Ro31-8220 partially restored tubulin levels (Figure 4E). Taken together, these results suggest that the potentiation of apicularen A-induced apoptotic cell death by PMA is associated with decreased tubulin protein levels.

## Discussion

The present study shows that PMA increases the cytotoxicity of apicularen A in HeLa cells. PKC inhibition completely blocked the synergistic effect of PMA on apicularen A-induced cytotoxicity and tubulin down-regulation. Specific knockdown of PKC isotypes revealed that PKCα is the unique mediator of that effect among PKC family members.

PMA induces apoptotic cell death in several kinds of cells [32,33]. In addition, the combination of PMA and anticancer drugs increases cytotoxicity [12,34], suggesting that PMA may be suitable as an anticancer agent within a drug combination regimen. In this study, although PMA alone did not induce cell death, it increased the cell death induced by apicularen A synergistically. This effect was completely blocked by the PKC inhibitor Ro31-8220, indicating that PKC is involved in the synergistic mechanism. Among PKC isotypes, PKCδ mainly promotes apoptosis [35]; however, since Ro31-8220 does not inhibit PKCδ [36], we had to consider that other PKC isotype(s) may be involved in the synergy. Specific knockdown of individual PKC isotypes demonstrated that PKCα is the mediator of the PMA-stimulated increase in apicularen A-induced apoptotic cell death in HeLa cells. Whether PKCα also mediates the synergistic effect of PMA with other anticancer drugs requires further investigation.

PMA is known to induce G_2_/M phase arrest in several cell lines [37,38], and PKCα activation is involved in the accumulation of cells in G_2_/M phase [38]. Our results show that the combination of PMA and apicularen A arrests cells in the G_2_/M phase, whereas exposure of cells to PMA alone only transiently increases the number of cells in the G_2_/M phase. The number of cells arrested in the G_2_/M phase in the presence of both PMA and apicularen A decreases in a time-dependent manner and leads to an increase in the number of cells in the sub-G1 phase. These results are consistent with previous findings showing that prolonged arrest in G_2_/M phase causes apoptotic cell death by blocking cell cycle progression [39].

We previously reported that apicularen A decreases tubulin protein levels and disrupts microtubule networks in human HM7 colon cancer cells by decreasing tubulin mRNA levels [5]. The present study reveals that apicularen A also promotes the disruption of microtubule networks through down-regulation of tubulin protein expression in HeLa cells, and that this phenomenon increased by PMA; however, PMA did not affect tubulin mRNA levels in the presence of apicularen A. The tubulin protein levels of cells exposed to the combination of PMA and apicularen A decreased slowly for 36 hours, and were decreased severely at the final time point (48 hours). Since a critical concentration of soluble tubulin is required for conservation of polymerized tubulin [40], and since PMA induces tubulin polymerization by regulating microtubule kinetics [41], it is possible that the microtubule polymerization induced by PMA may be initially resistant to apicularen A-induced tubulin down-regulation but finally collapses when soluble tubulin levels fall below the critical threshold required to support the polymerized networks. Since a decrease in tubulin protein levels and suppression of tubulin polymerization inhibit cell survival [42,43], lower tubulin protein levels in cells exposed to both PMA and apicularen A may explain the increased cytotoxicity observed in the presence of the two drugs. By contrast, since interference with microtubule dynamics decreases cell migration [44], the migration of cells exposed to the combination of PMA and apicularen A is expected to be lower than that of control cells; however, PMA regulates actin cytoskeleton reorganization and induces cell migration [45]. In addition, the combination of PMA and apicularen A did not change actin protein levels. Thus, we speculate that, although the combination of PMA and apicularen A induces G_2_/M phase arrest by disrupting microtubule networks, increased actin reorganization may contribute to increased cell migration (Additional file 1: Movie S1).

## Conclusions

In summary, this study shows that the combination of PMA and apicularen A synergistically induces apoptotic cell death in HeLa cells by mediating PKCα activation and disrupting intracellular microtubule networks. Since combination therapy is key to improving cancer treatment efficacy, these results may guide the development of novel therapeutic approaches to cancer.

## Competing interests

The authors declare that they have no competing interests.

## Authors’ contributions

KS Seo, JS Kim, JH Park, KS Song, and EJ Yun carried out the molecular studies and performed statistical analysis. YH Jung, JI Park, GR Kweon, and WH Yoon assisted in the interpretation of results. KS Seo, K Lim, and BD Hwang drafted and completed the manuscript. All authors read and approved the final manuscript.

## Pre-publication history

The pre-publication history for this paper can be accessed here:

http://www.biomedcentral.com/1471-2407/14/36/prepub

## Supplementary Material

Additional file 1: Movie S1 HeLa cells were exposed to 20 nM PMA in the presence or absence of 100 nM apicularen A for 48 hours. Time-dependent cell morphology was visualized using time-lapse video microscopy; control (upper left), PMA (upper right), apicularen A (lower right), combination of PMA and apicularen A (lower left). One second in the movie = 1 hour 18 minutes in real time. Click here for file
